# Neutral sphingomyelinase 2 modulates cytotoxic effects of protopanaxadiol on different human cancer cells

**DOI:** 10.1186/1472-6882-13-194

**Published:** 2013-07-27

**Authors:** Bonggoo Park, Yong-Moon Lee, Jae-Sung Kim, Youl Her, Ju Hee Kang, Seung-Hyun Oh, Hwan-Mook Kim

**Affiliations:** 1 College of Pharmacy, Gachon University, Incheon, Korea; 2 National Cancer Center, Gyeonggi-do, Goyang-si, Republic of Korea; 3 Department of Manufacturing Pharmacy, College of Pharmacy, Chungbuk National University, Cheongju, Korea; 4 Division of Biology of Aging, Department of Surgery, University of Florida, Gainesville, FL, USA; 5 BTGin Co. Ltd., Daejeon, Korea; 6 Current address: Department of Biochem and Mol Biol, College of Medicine, Korea University, GRL, Seoul, Korea

**Keywords:** Protopanaxadiol, Lipid raft, Neutral sphingomyelinase, Ceramide, Chemosensitization

## Abstract

**Background:**

Some of ginsenosides, root extracts from *Panax ginseng*, exert cytotoxicity against cancer cells through disruption of membrane subdomains called lipid rafts. Protopanaxadiol (PPD) exhibits the highest cytotoxic effect among 8 ginsenosides which we evaluated for anti-cancer activity. We investigated if PPD disrupts lipid rafts in its cytotoxic effects and also the possible mechanisms.

**Methods:**

Eight ginsenosides were evaluated using different cancer cells and cell viability assays. The potent ginsenoside, PPD was investigated for its roles in lipid raft disruption and downstream pathways to apoptosis of cancer cells. Anti-cancer effects of PPD was also investigated *in vivo* using mouse xenograft model.

**Results:**

PPD consistently exerts its potent cytotoxicity in 2 cell survival assays using 5 different cancer cell lines. PPD disrupts lipid rafts in different ways from methyl-β-cyclodextrin (MβCD) depleting cholesterol out of the subdomains, since lipid raft proteins were differentially modulated by the saponin. During disruption of lipid rafts, PPD activated neutral sphingomyelinase 2 (nSMase 2) hydrolyzing membrane sphingomyelins into pro-apoptotic intracellular ceramides. Furthermore, PPD demonstrated its anti-cancer activities against K562 tumor cells in mouse xenograft model, confirming its potential as an adjunct or chemotherapeutic agent by itself *in vivo*.

**Conclusions:**

This study demonstrates that neutral sphingomyelinase 2 is responsible for the cytotoxicity of PPD through production of apoptotic ceramides from membrane sphingomyelins. Thus neutral sphingomyelinase 2 and its relevant mechanisms may potentially be employed in cancer chemotherapies.

## Background

Ginsenosides, root extracts from ginseng have been used in many countries for treatment and prevention of a variety of cancers of stomach, lung, liver, pancreas, colon and so on, since they are responsible for most of ginseng’s pharmacological and biological effects [1]. Ginsenosides suppress proliferation and angiogenesis of cancer cells, reduce malignant transformation, and increase cell deaths due to apoptosis and necrosis [2,3]. They can also be used in combinations with conventional chemotherapeutic agents for treatment of cancers and prevention of their developments in advance.

Recent investigations have suggested that specialized subdomains of cell membrane termed lipid rafts are actively involved in regulation of essential cellular processes such as cell proliferation, differentiation and apoptosis, many of which are altered during development of various tumors [4,5]. Lipid rafts are enriched in cholesterol, sphingomyelin, gangliosides, caveolins, flotillins, etc. Depending on their lipid and protein compositions, lipid rafts are classified into caveolae and non-caveolar ones [4]. Caveolar lipid rafts are flask-shaped membrane invaginations enriched in cholesterol and caveolins, proteins from caveolin family, while non-caveolar ones are associated with proteins of reggie family, flotillin-1 and flotillin-2 [4]. Due to its high concentrations in lipid rafts, long and saturated sphingolipids make microdomains physicochemically distinct from the remaining of plasma membranes abundant in short and unsaturated phospholipids through their closer interactions with cholesterols [4]. Cholesterol depleting agents such as methyl-β-cyclodextrin (MβCD) can remove cholesterols out of lipid rafts, changing their physical properties and thereby modifying lipid raft-dependent signaling pathways in vital cellular processes [6]. Ginsenosides, phytosterols from *Panax ginseng*, are natural cholesterol derivatives with hydroxyl groups and oligosaccharide moieties, so many investigators have studied effects on which these ginsenosides might have against crucial functions of lipid rafts.

The ginsenoside Rh2 has been known to increase membrane fluidity and induce apoptosis via lipid raft disruption and thereby caspase-8 dependent Fas activation [7]. Rh2 also reduces the levels of lipid rafts in plasma membranes by increasing their internalization through unknown mechanisms, leading to Akt inactivation and ensuing apoptosis [8]. 25-hydroxyprotopanaxadiol (25-OH-PPD), another ginsenoside from *Panax ginseng* demonstrated a potent cytotoxicity against a variety of prostate cancer cells, compared to its related ginsenosides [9]. Anti-cancer effects against both prostate and lung cancer cells were repeated with 25-OCH3-protopanaxadiol (25-OCH3-PPD) from *Panax notoginseng in vitro* and *in vivo*[10,11]. These ginsenosides modulated protein regulators in cell proliferation and apoptosis with relatively low toxicity against non-cancer cells. Like Rh2 inhibiting Akt signaling in lipid rafts in favor of apoptosis, 20S-protopanaxadiol (aPPD), another PPD derivative significantly inhibited Akt activities only in lipid rafts with no changes in total Akt levels of plasma membranes [12].

In present study, we have tested 8 ginsenosides from *Panax ginseng* for their cytotoxicity using different cancer cell lines. We have demonstrated that cytotoxic effects of PPD on cancer cells were mediated through production of intracellular ceramides from membrane sphingomyelins. Sphingolipids such as sphingomyelins have biologically active roles in cellular functions such as growth and differentiation through production of ceramides, signaling molecules rather than structural elements in cell membrane. In light of the fact that accumulation of intracellular ceramides appeared to be important in chemosensitization of cancer cells during chemotherapies using multiple drugs [13], potential of the natural product as chemotherapeutic agent are further discussed in terms of chemosensitization of cancer cells to apoptosis. Finally, PPD demonstrated its *in vivo* anti-tumor activity in mouse xenograft tumor model, confirming its *in vitro* effects on cancer cells.

## Methods

### Cell lines and reagents

Cancer cells were obtained from American Type Culture Collection (Rockville, MD, USA) and cultured in DMEM or RPMI-1640 medium (Gibco BRL, Grand Island, NY, USA) supplemented with 10% fetal bovine serum (Hyclone, Logan, UT, USA), 2 mM L-glutamine and 1% penicillin/streptomycin (Gibco BRL) at 37°C with 5% CO2. Ginsenosides, including PPD (purity > 98%) were obtained from BTGin (Daejeon, Korea). Propidium Iodide (PI) solution was obtained from Sigma-Aldrich (St. Louis, MO, USA).

### Cell viability assay

In XTT assay using Cell Proliferation Kit II (XTT) (Roche Applied Science, Mannheim, Germany), cells were plated into 96 well plates at 0.3 – 1.0 ×10^4^ cells per well overnight and treated with PPD or/and other compounds for 24 or 48 hr. After addition of XTT labeling mixture for 2 hr at 37°C, absorbance was measured at 490 nm with a reference wavelength at 650 nm. In Sulforhodamine B (SRB) assay described previously [14], after treatment with various ginsenosides for 24 or 48 hr, cells were added with 50 μl 50% trichloroacetic acid (TCA), fixed for 2 h at 4°C, washed with tap water 3 times and air-dried. TCA-fixed cells were stained for 30 min with SRB (0.4% in 1% acetic acid), followed by washing with 1% acetic acid and air-dried. After addition of 100 μl 10 mM Tris, pH 10.5, absorbance was measured at 570 nm. In WST-1 assay with EZ-Cytox Cell Viability Assay kit (Daeillab Service, Seoul, Korea), after incubation with various ginsenosides for 24 or 48 hr, cells were added with 10 μl WST-1 reagent, then incubated for 15 – 60 min at 37°C. Absorbance was determined at 460 nm with microtiter plate reader.

### Cell cycle analysis

K562 cells were plated into 6 well plates at 3 × 10^5^ cells per well overnight, then treated with PPD at specified concentrations for 48 h. In chemosensitization experiments, cells were treated with PPD or Cisplatin with or without doxorubicin for 48 hr at indicated concentrations. After treatments, cells were harvested, washed with PBS, fixed in 70% ethanol overnight at -20°C, stained with propidium iodide overnight at 4°C, then analyzed using FACSCalibur flow cytometer (BD Biosciences, San Jose, CA, USA).

### Western blotting

Whole cell proteins were separated in 8-15% SDS polyacrylamide gel, transferred to PVDF membranes (Pall Corporation, Pensacola, FL, USA) and probed with anti-Cdk2, anti-Cdk4, anti-caspase-9, anti-β-actin and anti-IGF-1R antibodies (Santa Cruz Biotechnology, Santa Cruz, CA, USA), and anti-Cdk6, anti-Cyclin A, anti-Cyclin B1, anti-α-tubulin, anti-poly (ADP-ribose) polymerase (PARP), anti-Bcl-2, anti-pAkt (Ser473), anti-caspase-8 and anti-Bid (Cell Signalling Technology, Beverly, MA, USA) overnight at 4°C. Next day, membranes were incubated with horseradish peroxidase (HRP)-conjugated goat anti-mouse IgG, bovine anti-goat IgG and bovine anti-rabbit IgG (Santa Cruz) and detected with enhanced chemiluminescent (ECL) detection system (Amerisham Biosciences, Little Chalfont, United Kingdom).

### Cell imaging

After treatment with PPD overnight as described above, K562 cells were centrifuged and treated with 2 μM BODIPY-C12-SM and 2 μM BODIPY-Cholesterol ester (Invitrogen, Grand Island, NY, USA) respectively in RPMI medium for 30 min at 4°C to label sphingomyelin and cholesterol. After staining, cells were washed with PBS twice and placed onto confocal stage to collect images.

### Mass spectrometry

K562 cells were seeded into petri dishes at 1 × 10^6^ cells per dish overnight, then treated with PPD at specified concentrations for 6 or 16 hr. After treatments, cells were harvested, washed twice with ice-cold PBS and extracted with extraction buffer (isopropanol: water: ethyl acetate = 30:10:60), as described previously [15]. Amounts of ceramides were determined with LC/MS/MS system (LC, Thermo surveyor LC pump plus, American Laboratory Trading, East Lyme, CT, USA; MS, LCQ Fleet, Thermo Fisher Scientific, Waltham, MA, USA) using C-17 ceramide as an internal standard.

### Inhibition and siRNA interference

In inhibition studies, cells were plated into 96 well plates at 0.3 – 1.0 ×10^4^ cells per well overnight, preincubated for 1 hr with neutral sphingomyelinase 2 (nSMase 2) inhibitor GW4869 at 10 μΜ (Sigma-Aldrich), then treated with PPD at specified concentrations for 48 h. Two nSMase 2 siRNAs were purchased from Bioneer (Daejeon, Korea): siRNA 1141725 sense: GCU ACU UCG AGU ACA UCC U and antisense AGG AUG UAC UCG AAG UAG C and siRNA 1141726 sense: CAC GAA CGG CCU GUA CGA U and antisense: AUG GUA CAG GCC GUU CGU G. Scrambled siRNA (SCR) (Bioneer) was used as negative control: sense CCU ACG CCA CCA AUU UCG U and antisense: ACG AAA UUG GUG GCG UAG G. Real-time PCR was performed to quantify mRNA expressions of nSMase 2 after knockdown with siRNA 1141725 and 1141726 (Additional file 1). Total RNA was isolated from cultured cells using Trizol (Invitrogen). cDNAs were synthesized using oligo (dT) and SuperScript III reverse transcriptase (Promega, Madison, WI, USA), then amplified using iQ SYBR Green PCR Master Mix (Bio-Rad Laboratories, Hercules, CA, USA) in conjunction with Mx3005P QPCR Systems (Agilent Technologies, Santa Clara, CA, USA). Primer sets were as follows: nSMase 2 qPCR primer P109289 (Bioneer); glyceraldehyde-3-phosphate dehydrogenase, 5′-TGGTATCGTGGAAGGACTCATGAC-3′ (forward) and 5′-ATGCCAGTGAGCTTCCCGTTCAGC -3′ (reverse). Fold changes were calculated after normalization to endogenous GAPDH, using comparative 2-^ΔΔCt^ method [16]. In transfections, cells were cultured in complete medium, seeded into 96 well plate at 0.5 – 1.0 × 10^4^ cells per well using culture media without antibiotics overnight along with mixture of Lipofectamine (Invitrogen) and SCR or siRNAs (Bioneer) at 10 nM. Next day, transfected cells were treated with various concentrations of PPD for 48 hr, then were assayed for their viabilities using WST-1, as described above.

### Mouse xenograft tumor model

All animal care and experimental procedures were approved by Institutional Animal Care and Use Committee (IACUC) at Gachon University in Incheon, Korea. In mouse xenograft tumor model, female BALB/c nude mice (Charles River, Wilmington, MA, USA) were subcutaneously injected with K562 cells to establish xenografted tumors and provided with food and water ad libitum under standard light/dark cycles. When K562 tumor sizes reached 47.6 mm^3^, xenografted mice were randomly assigned into 2 groups (n = 4 mice per group) to receive daily intravenous injections with vehicle, Cisplatin (5 mgkg^-1^) or PPD (25 or 50 mg kg^-1^ body weight) 5 days per week over 2 weeks. Tumor volumes were measured with calipers over 10 days as well as body weights and were calculated using formula (width^2^ × length) 2^-1^, where width is the smaller tumor diameter. Mice were killed on the 11^th^ day and their tumor weights were measured.

### Statistical analysis

The values in figures were expressed as means ± s.d. Unpaired t tests were used to compare two groups. Values of P < 0.05, P < 0.01 and P < 0.001 were considered as statistically significant.

## Results

### Protopanaxadiol exhibits the most potent cytotoxic effects against a variety of cancer cells among 8 ginsenosides tested in cell survival assays

**Figure 1:**
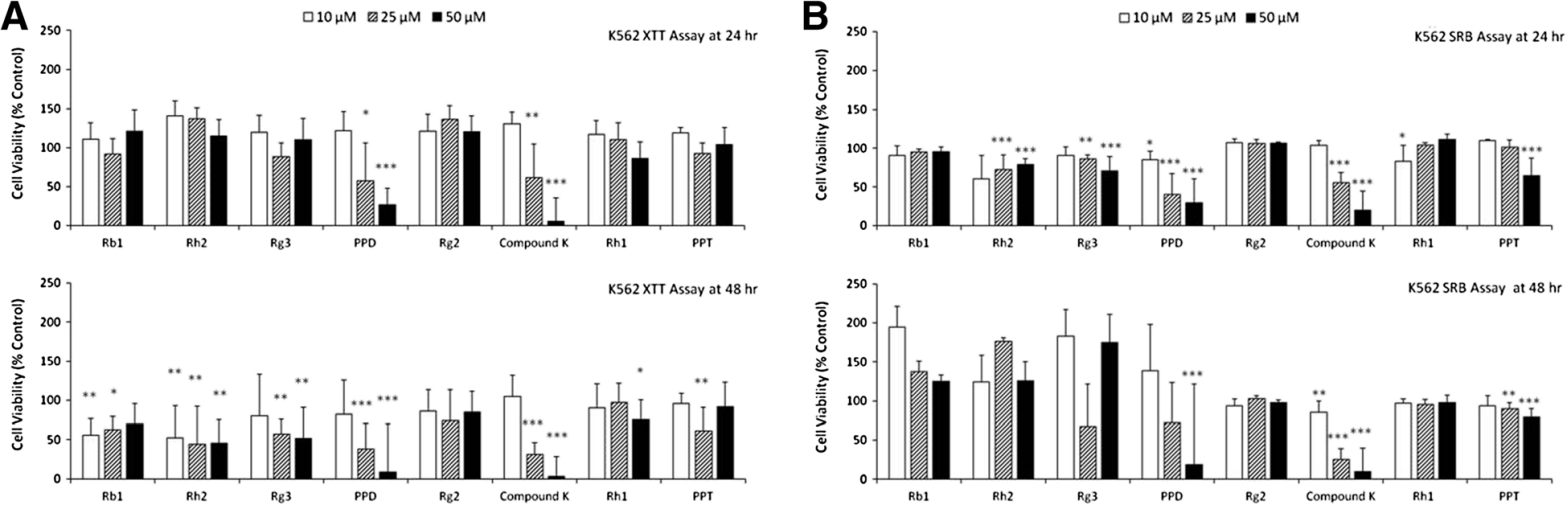
**Protopanaxadiol (PPD) exhibited the most potent cytotoxic effects on cancer cells of various malignancies.** K562 cells were treated with PPD at 0, 25 or 50 μM for 24 or 48 hr, then their viabilities were assessed using XTT **(A)** and SRB assays **(B)**. *, p < 0.05; **, p < 0.01; ***, p < 0.001 for control (vehicle) are considered as significant.

**Figure 2:**
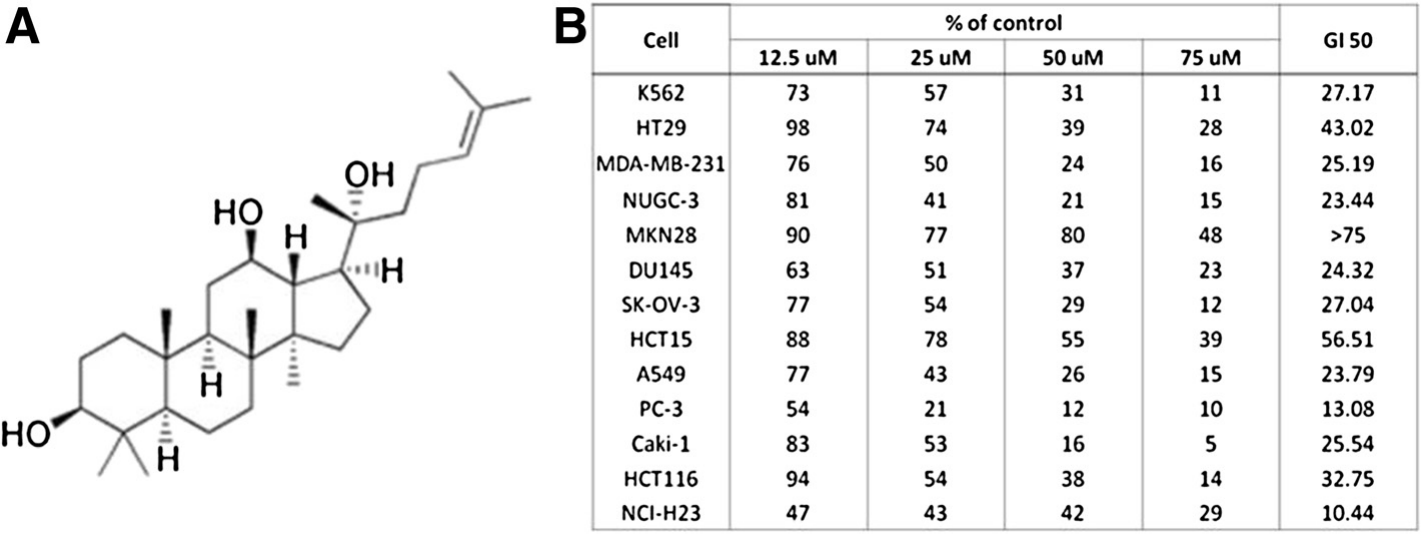
**A variety of cancer cells showed high to moderate sensitivities to PPD treatment. (A)** Structure of PPD. **(B)** GI_50_’s of PPD was calculated against 13 cancer cell lines. GI_50_ is the concentration that inhibits cell growth by 50%.

Eight ginsenosides extracted from *Panax ginseng* were evaluated for their *in vitro* cytotoxic activities against 5 human cancer cell lines (Figure 1, Additional files 2 and 3), using 2 different cell viability assays. Most of cell viability assays measure specific components of live cells, so results could be varied depending on each assay method. Here, we employed XTT and SRB assays to more convincingly examine effects of 8 ginsenosides on various cancer cells. SRB assay measures total biomass, namely number of cells, by staining cellular proteins with Sulforhodamine B, whereas dye reduction by mitochondrial reductants on cell surface is determined in XTT assay, actually measuring pyridine nucleotide redox status of live cells [14,17]. We used 5 human cancer cell lines; NCI-H23 lung cancer cells, PC-3 prostate cancer cells, ACHN and Caki-1 renal cancer cells, and K562 leukemia cells, since ginsenosides might have cell type- or tissue-specific cytotoxic effects. PPD significantly reduced viabilities of 5 different cancer cells at various concentrations for 24 or 48 hours as well as compound K both in dose- and time-dependent ways (Figure 1, Additional files 2 and 3). PPD is a tetracyclic triterpene saponin which has a 4 ring backbone structure and 3 hydroxyl groups attached with structural resemblance to cholesterol (Figure 2A) [1]. Cell survival assays using additional cancer cell lines of different malignancies were employed to quantitatively measure GI_50_′s of PPD against a variety of cancer cells, which are the concentrations of PPD to inhibit cell survival by 50% (Figure 2B). Most of cancer cell lines tested exhibited their GI_50_ values between 20 μM and 50 μM with a couple of exceptions. Colon cancer cells (HT29, HCT116 and HCT15) and gastric cancer cells (MKN28) appeared to be more resistant to PPD than other cancer cells such as K562 (leukemia), NCI-H23 and A549 (lung), Caki-1 (renal), PC-3 and DU145 (prostate), MDA-MB-231 (breast), NUGC-3(gastric), SK-OV-3 (ovarian), indicating that anti-cancer activities of PPD might be cell type- or tissue-specific (Figure 2B).

### PPD inhibits cell proliferation through modulation of cell cycle regulators as well as apoptotic proteins, leading to cell death

**Figure 3:**
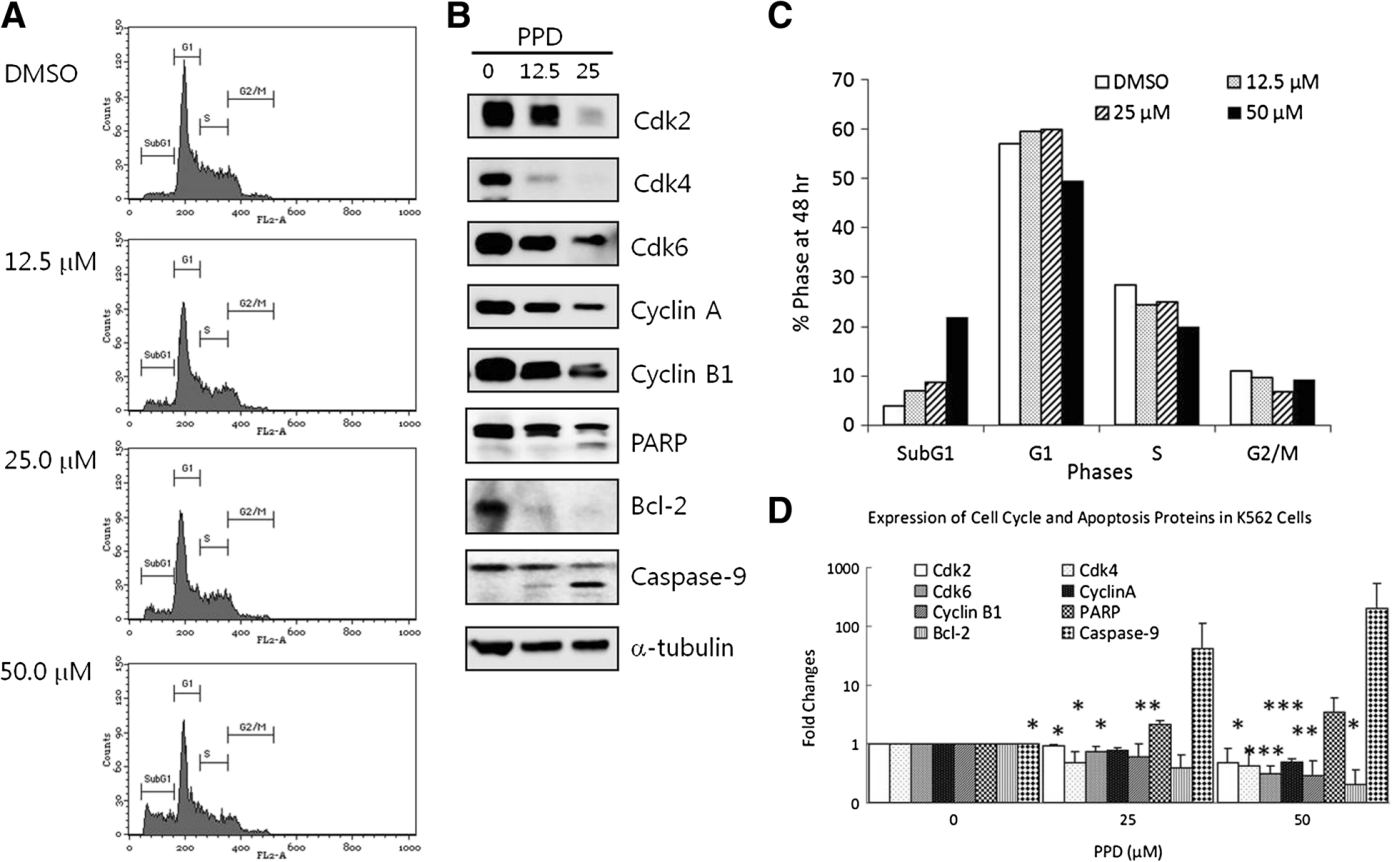
**Effects of PPD on cell cycle progression of cancer cells were analyzed using flow cytometry. (A)** K562 cells were exposed to PPD at 0, 12.5, 25 and 50 μM for 48 hr. Then, they were fixed with ethanol, stained with propidium iodide and analyzed using flow cytometry. **(B)** K562 cells were exposed to PPD at 0, 12.5 and 25 μM for 24 hr, then expression levels of cell cycle regulators and pro-apoptotic proteins were determined with Western blotting using anti-cdk2, -cdk4, -cdk6, -cyclinA and -cyclinB1 antibodies, -PARP, -Bcl-2, and -caspase-9 antibodies. **(C)** Relative percentages of cells were calculated at sub-G_1_, G_1_, S and G_2_/M phases. **(D)** All experiments were repeated three times with similar results, so the means ± standard deviations of band intensities were calculated in our Western blot analyses for cell cycle and apoptosis components. *, p < 0.05; **, p < 0.01; ***, p < 0.001 for control (vehicle) are considered as significant.

In cell cycle analysis using flow cytometry, PPD arrested cell cycle in SubG1 phase, which contains mostly dead cells (Figure 3A and C). Significant numbers of cells from G1, S and G2/M phases turned into fragmented dead cells of subG1 phase in dose-dependent way, as PPD concentration increased up to 50 μM. Thus, cell cycle regulators such as cyclins and cyclin dependent kinases (cdks) are likely to be modulated by PPD as well as proteins in cell death pathways. Cdk2 regulates cell cycle during G1 to S and S to G2/M phase transitions through its interaction with cyclin A or E, while cdk4 and cdk6 are essential for cell cycle progression during G1 phase and G2 to M transition respectively in concert with cyclin D. In agreement with cell cycle analysis, the aforementioned cdks and cyclins were downregulated in Western blot analysis of PPD treated K562 cells (Figure 3B and D). In addition, cleaved active form of caspase-9 was increased as well as cleaved caspase-3 substrate PARP, while anti-apoptotic protein Bcl-2 was downregulated, indicating that apoptotic proteins are also modulated by PPD (Figure 3B and D).

### The ginsenoside mediates anti-cancer activities by disrupting lipid rafts in plasma membrane

**Figure 4:**
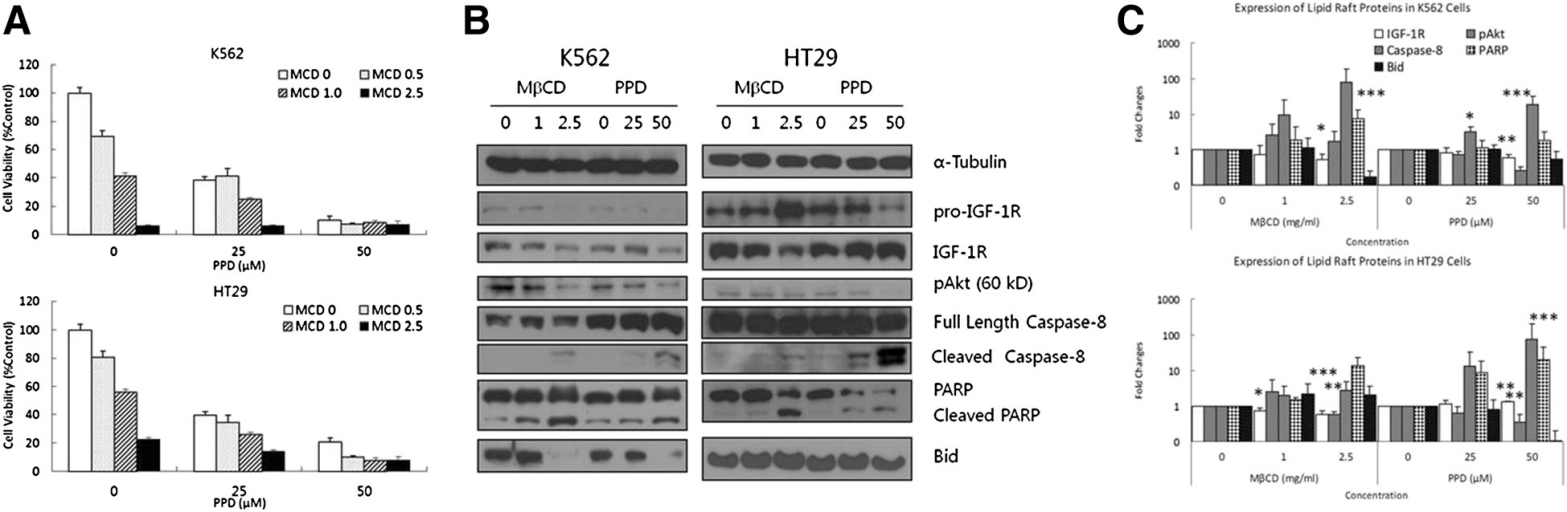
**PPD disrupted lipid rafts in plasma membranes of cancer cells through cell type specific mechanisms. (A)** K562 and HT29 cells were exposed to PPD at 0, 25 and 50 μM in presence of 0, 0.5, 1.0 or 2.5 mg/ml MβCD for 24 hr. The cell viabilities were measured using WST-1 assay. **(B)** K562 and HT29 cells were exposed to MβCD at 0, 1.0 or 2.5 mg/ml and PPD at 0, 25 or 50 μM for 24 hr, then expression levels of lipid raft-associated proteins were determined with Western blotting using anti-IGF-1R, -pAkt, -caspase-8, -PARP, and -Bid antibodies. **(C)** All experiments were repeated three times with similar results, so the means ± standard deviations of band intensities were calculated in our Western blot analyses for lipid raft-associated proteins. *, p < 0.05; **, p < 0.01; ***, p < 0.001 for control (vehicle) are considered as significant.

We investigated the possibility that PPD may disrupt lipid rafts as one of cytotoxic mechanisms like Rh2 and aPPD [7,8,12]. K562 and HT29 cells were treated with PPD with or without MβCD, another lipid raft disrupter [6]. MβCD minimally enhanced cytotoxic effects of PPD against both cancer cells, indicating that PPD might act through common mechanisms with those of MβCD despite possible distinct pathways (Figure 4A). As shown in Figure 4B and C, PPD and MβCD modulate lipid raft-dependent signaling pathways including IGF-1R, pAkt, PARP, Caspase-8 and Bid in two different cancer cells. Interestingly, two lipid raft disrupters differentially modulated IGF-1R and Bid, although they exhibited similar expression patterns in the remaining lipid raft proteins under investigation. Both MβCD and PPD decreased levels of IGF-1R as well as Bid in K562 cells, while IGF-1R and Bid were upregulated by PPD and MβCD respectively in HT29 cells, suggesting that cytotoxicity of PPD and MβCD is cell type-specific (Figure 4B and D).

### PPD exerts its cytotoxic effects on cancer cells through upregulation of neutral sphingomyelinase 2

**Figure 5:**
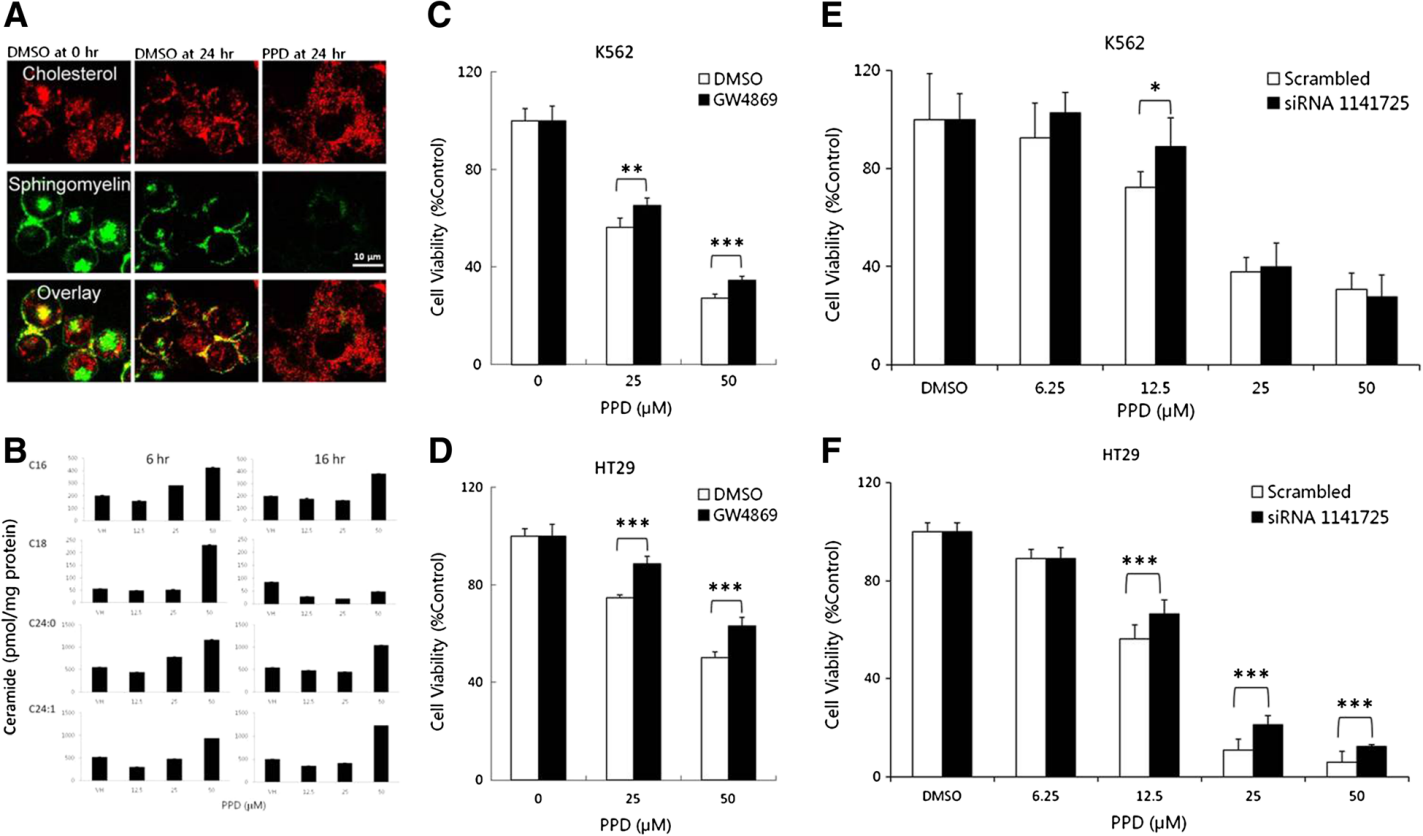
**PPD exerted its cytotoxic effects on cancer cells through activation of neutral sphingomyelinase 2. (A)** K562 cells were exposed to PPD at 0 (DMSO) and 25 μM for 24 hr. They were stained with BODIPY-Cholesterol ester or BODIPY-C12-SM, then examined using confocal microscopy. **(B)** K562 cells were exposed to PPD at 0, 12.5, 25 and 50 μM for 6 or 16 hr, then levels of different ceramides were analyzed using LC-MS/MS. K562 **(C)** and HT29 **(D)** cells were preincubated with neutral sphingomyelinase (nSMase) inhibitor GW4869 for 1 hr, exposed to PPD at 0, 25 and 50 μM for 48 hr and evaluated for cell viabilities using WST-1 assay. Inhibition studies with GW4869 were repeated three times with similar results. K562 **(E)** and HT29 **(F)** cells were transfected with nSMase 2 siRNA 1141725, exposed to PPD at 0, 6.25, 12.5, 25 and 50 μM for 48 hr and evaluated for cell viabilities using WST-1 assay. Inhibition studies with siRNA transfections were repeated twice with similar results. *, p < 0.05; **, p < 0.01; ***, p < 0.001 for control (vehicle) are considered as significant.

We hypothesized that PPD might have common mechanism(s) among cell type-specific cytotoxic effects in distinct cancer cells. To this end, we visualized cholesterols and sphingomyelins rich in the microdomains with fluorescent sphingomyelin and cholesterol (Figure 5A). PPD treatment removed sphingomyelins out of cell membranes without any significant reduction of membrane cholesterols (Figure 5A), suggesting that the ginsenoside might modulate signaling pathways through lipid rafts differently from MβCD [8]. To determine the fate of sphingomyelins disappearing in plasma membrane, we measured intracellular levels of various ceramides, the immediate degradation products of sphingomyelins after PPD treatments for 6 and 16 hr, using mass spectrometry (Figure 5B). Levels of ceramides in cell lysates were significantly increased in dose-dependent way as PPD concentration reached 50 μM both at 6 and 16 hr. The production of intracellular ceramides induces apoptosis in different cancer cells, thus increased levels of cellular ceramides are likely to be responsible for the reduced survival of PPD-treated K562 and HT29 cells [18,19]. Then, we hypothesized that PPD induced cancer cell deaths could be reversed by knockdown or inhibition of sphingomyelinase, which degrades membrane sphingo-myelins into ceramides. To this end, we knocked down neutral sphingomyelinase 2 with si-RNA transfection or inhibited its enzyme activity with an inhibitor GW4869 (Figure 5C - F). Blockage of the responsible neutral sphingomyelinase 2 decreased PPD-induced cell deaths in K562 and HT29 cells to significant extent. Therefore, these results demonstrate that PPD converts membrane sphingomyelins into intracellular ceramides through activation of neutral sphingomyelinase 2 as one of its major cytotoxic mechanisms.

### PPD greatly sensitizes cancer cells to cell death by anti-cancer drugs such as Doxorubicin

**Figure 6:**
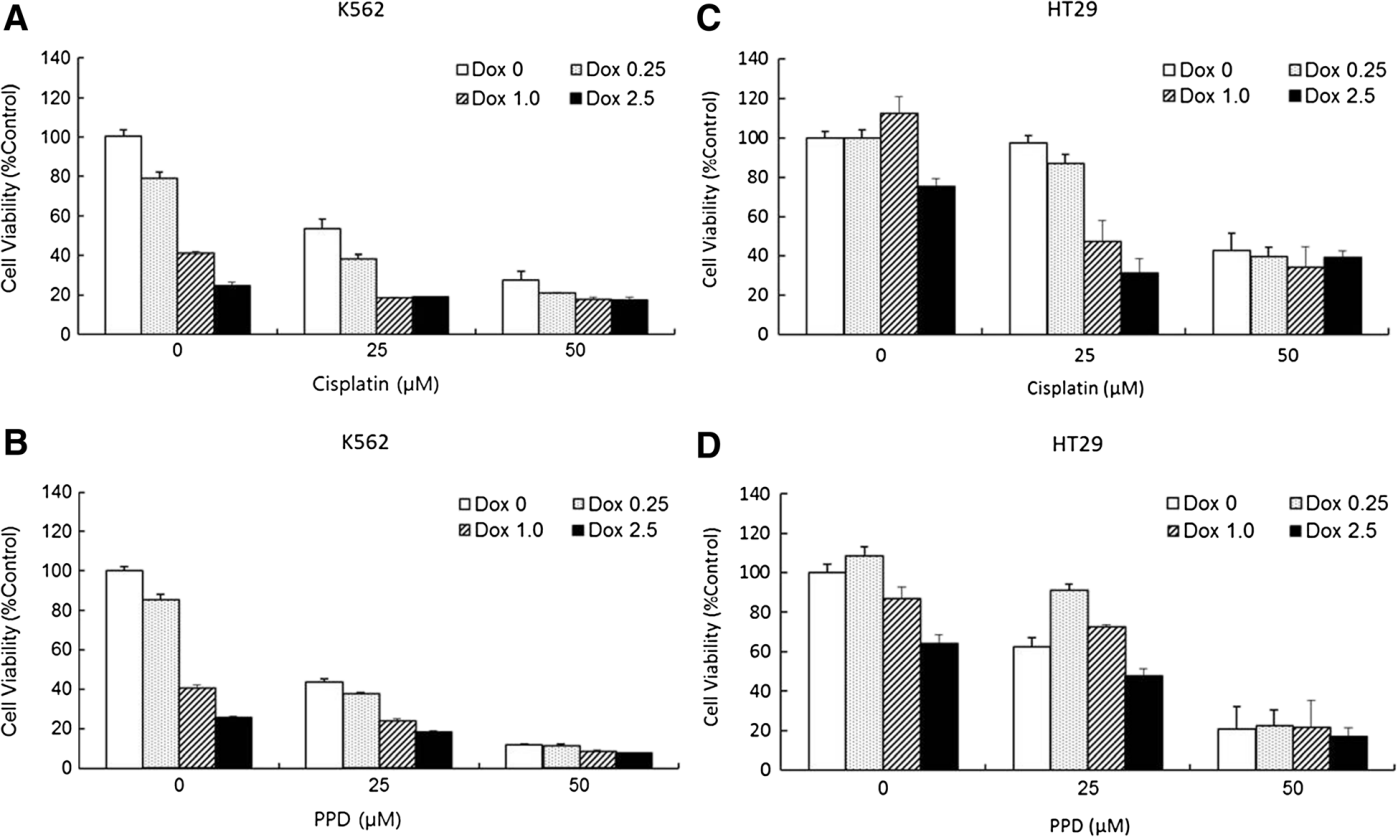
**PPD sensitized cancer cells to doxorubicin treatment.** K562 **(A** and **B)** and HT29 **(C** and **D)** cells were exposed to Cisplatin **(A** and **C)** or PPD **(B** and **D)** at 0, 25 and 50 μM in presence of 0, 0.25, 1.0 or 2.5 μM Doxorubicin for 48 hr. All experiments were repeated three times with similar results.

**Figure 7:**
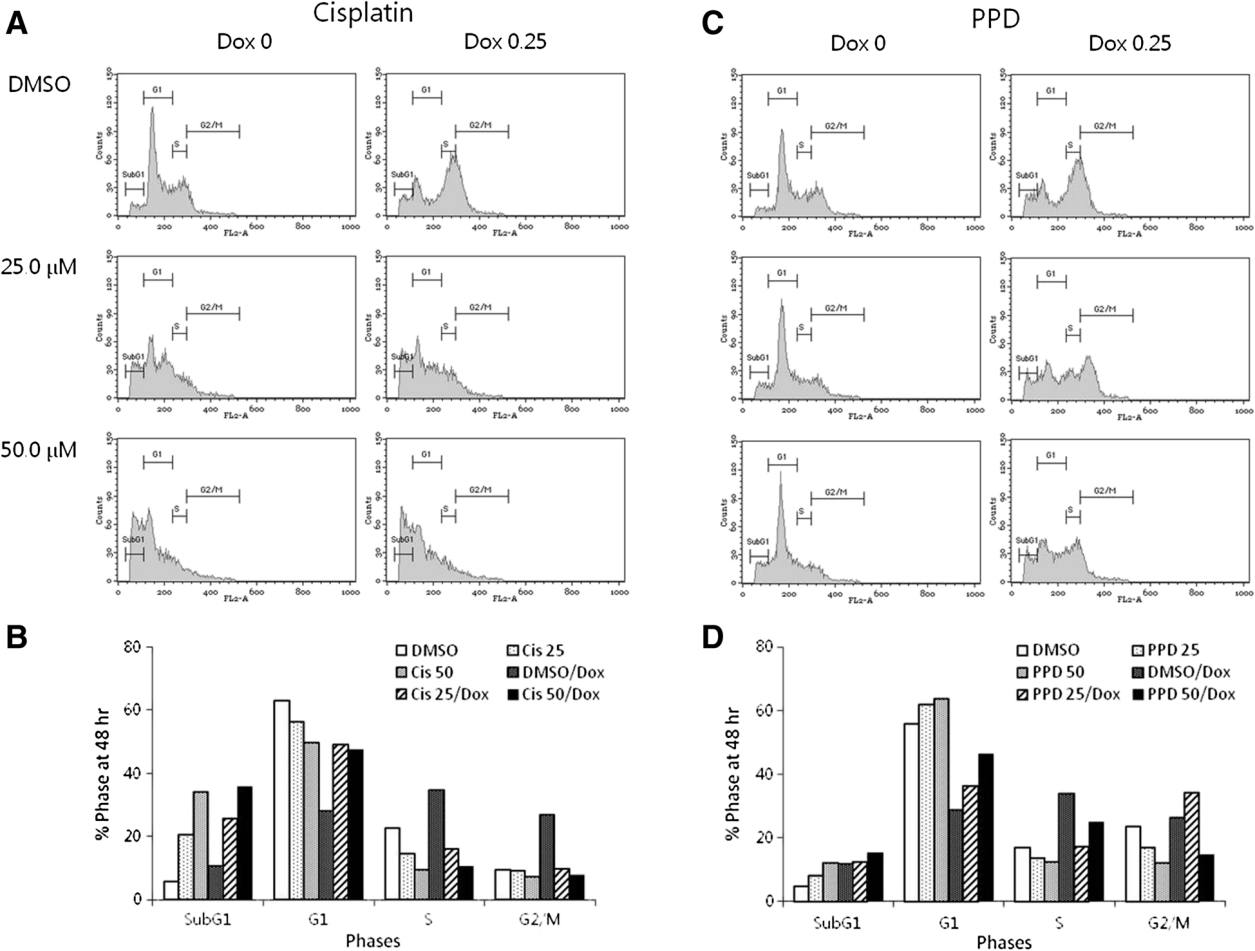
**Sensitization by PPD is mediated through altering cell cycle progression and apoptosis.** K562 cells were exposed to Cisplatin **(A** and **B)** or PPD **(C** and **D)** at 0, 25 or 50 μM with or without 0.25 μM Doxorubicin for 48 hr. Then they were fixed with ethanol, stained with propidium iodide and analyzed using flow cytometry. All experiments were repeated three times with similar results.

Previous studies have shown that endogenous and cell permeable exogenous ceramides demonstrated pro-apoptotic activities against a variety of cancer cells [18-20]. Thus, biologically active ceramides have gotten a great deal of attentions in cancer chemotherapeutics in that they could be utilized as potential targets to enhance effectiveness of conventional anti-cancer drugs such as doxorubicin in a synergistic way [21]. Here, we demonstrated that PPD greatly sensitizes K562 and HT29 cells to doxorubicin to similar extent where Cisplatin does in additive or synergistic ways, depending on different combinations of their concentrations (Figure 6A-D). Although there were some differences in sensitivities of two cell types, PPD induced chemosensitization seemed dose-dependent but not cell type-specific. Thus our protopanaxadiol could be an effective adjunct to chemotherapies using highly toxic anti-cancer drugs such as Doxorubicin through their reduced usages, although complete molecular mechanisms of additive or synergistic effects remain to be determined. PPD enhanced sensitivities of cancer cells to Doxorubicin through the increased growth inhibition and apoptosis just like Cisplatin does, as shown in cell cycle analyses using flow cytometry (Figure 7A-D).

### The ginsenoside exhibits in vivo anti-tumor activities against xenografted K562 cancer cells

**Figure 8:**
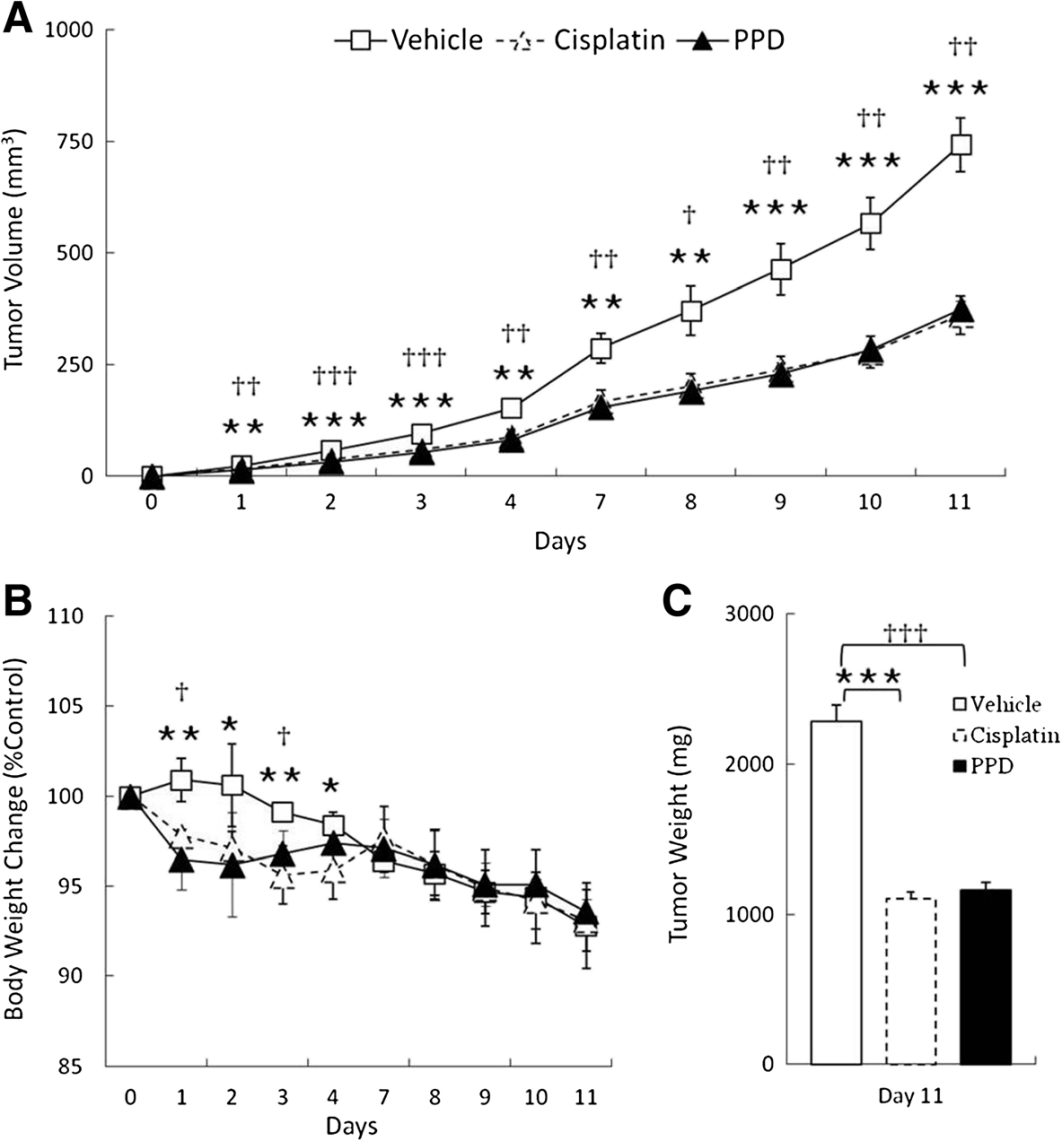
**PPD reduced both tumor volumes and weights in *in vivo *xenograft mouse model.** K562-xenografted BALB/c nude mice were randomly divided and treated daily with vehicle, Cisplatin (5 mg/kg) or PPD (25 or 50 mg/kg), when their tumor volumes reached 47.6 mm^3^. **(A)** The tumor volumes were determined using the formula (width^2^ × length)/2, where width represents the smaller diameter. **(B)** The body weights were measured daily during treatments. Body weight changes were given as percentage of mouse body weights on day 0. **(C)** The tumor weights were measured on the final 11^th^ day. Unpaired Student’s *t* test was used for comparisons between control (vehicle) and Cisplatin or PPD group. *, p < 0.05; **, p < 0.01; ***, p < 0.001 for control (vehicle) and Cisplatin, and †, p < 0.05; ††, p < 0.01; †††, p < 0.001 for control (vehicle) and PPD are considered as significant.

We next tested the potent *in vitro* cytotoxicity of PPD against cancer cells *in vivo* using mouse xenograft tumor model. K562-xenografted tumors on BALB/c nude mice were treated daily with vehicle, Cisplatin (5 mg kg^-1^ body weight) or PPD (25 or 50 mg kg^-1^ body weight), once their sizes reached 47.6 mm^3^. Both PPD and Cisplatin started reducing tumor volumes compared to control group from the first day after drug treatments, with total tumor volumes decreased by 51.7% with Cisplatin and by 49.2% with PPD on the 11th day (Figure 8A). However, weight losses in PPD-treated mice were less severe than in Cisplatin-treated ones during the early days, suggesting that PPD might be rather safer as a novel chemotherapeutic agent, although both PPD and Cisplatin didn’t induce any significant changes in mouse body weights later despite their potent anti-tumor activities (Figure 8B and C).

## Discussion

Novel therapeutic agents, giving better efficacy but less toxicity and resistance, have been in great need to treat a variety of cancers refractory to our current cancer therapeutics, thereby resulting in poor outcomes even after intensive treatments. More than 30 ginsenosides identified from ginseng extracts largely consist of four ring structures of 17 carbons derived with multiple sugar moieties [1]. Despite variable effectiveness, their potential as an adjunct to cancer therapeutics has been investigated for a long time due to their potent cytotoxicity against cancer cells but still low toxicity on non-cancer cells. In present investigation, the potent protopanaxadiol illustrated multifaceted aspects of its cytotoxicity through modulation of multiple cell cycle regulators and cell death proteins in cell cycle analyses.

Although PPD disrupts lipid rafts through certain common mechanisms with cholesterol depletion by MβCD, some PPD-specific modulation of essential signaling path-ways seemed to exist in lipid rafts. Previous studies have demonstrated that some lipid raft-associated signaling proteins are modulated by other ginsenosides such as Rp1, aPPD and Rh2 [7,8,12,22]. Rp1 inhibited proliferation of human breast cancer cells such as MCF-7 and MDA-MB-231 through suppression of IGF-1R/pAkt pathway, pAkt of which is also suppressed by Rh2 and aPPD [5,8,12,22]. Activation of IGF pathway is a crucial prerequisite to malignant transformation during development of various cancers as well as differentiation of normal cells such as adipocytes [23,24]. IGF-1R is overexpressed in most cancer cells, so it’s not surprising that some tumor suppressors exert anti-cancer activity through transcriptional suppression of IGF-1R gene [25]. In Western blot analysis, IGF-1R was downregulated by MβCD and PPD in K562 cells, whereas surprisingly, PPD upregulated IGF-1R in HT29 cells, suggesting that IGF-1R might have a minor role in cytotoxicity of PPD on HT29 cells due to other potent cytotoxic mechanisms. Another ginsenoside Rg1 attenuated cytotoxic effects of neurotoxin 6-OHDA on human neuroblastoma cells through IGF-1R receptor signaling [26], indicating that ginsenoside-mediated IGF-1R signaling is cell-type specific depending on agents used. Interestingly, MβCD also had a cell-type specific effect in K562 and HT29 cells, since Bid was upregulated in HT29 cells despite its lower level in K562 cells. Since lipid rafts are essential for cancer development, ability of PPD to disrupt the microdomains can be employed as a chemotherapeutic target for cancer treatments, as shown in other ginsenosides such as aPPD and Rh2 [7,12]. In addition, Rh2-induced Fas activation and its synergism with betulinic acid toward apoptosis of cancer cells through caspase-8 activation made this lipid raft disruptor an adjunct candidate for anti-cancer therapies [7,27].

Intracellular levels of ceramides are modulated through hydrolysis of sphingomyelins by sphingomyelinases, *de novo* synthesis involving ceramide synthases and production of pro-survival molecule, sphingosine 1-phosphate from ceramide by sphingosine kinases [28]. Both neutral sphingomyelinases (nSMases) and acid sphingomyelinases (aSMases) mediate formation of ceramides from sphingo-myelins in response to apoptotic inducers including chemotherapeutic agents [18,20,29]. In our efforts to identify PPD-specific cytotoxic mechanisms in lipid rafts, we demonstrated that accumulated intracellular ceramides mediate cytotoxic effects of PPD, leading to growth inhibition and apoptosis in distinct cancer cells. Since knockdown or inhibition of neutral sphingomyelinase 2 reduced PPD induced cytotoxic effects against cancer cells to significant extent, this enzyme must be at least partially responsible for growth arrest and apoptosis of cancer cells through increasing intracellular ceramide levels. A recent publication has shown that Withanolide D, an herbal compound from *Withania Somnifea*, also activated neutral sphing-omyelinase 2/ceramide pathway, inducing apoptosis in leukemia cells [19]. To our knowledge, we have demonstrated for the first time that PPD, one of ginsenosides from *Panax ginseng*, mediates its cytotoxicity against cancer cells through production of intracellular ceramides by upregulation of neutral sphingomyelinase 2.

We also demonstrated that PPD could significantly enhance Doxorubicin induced cell deaths in two different cancer cell lines as an effort to reduce the usage of toxic anti-cancer drugs. Cisplatin activates acid sphingomy-elinase, followed by production of ceramides, leading to CD95 redistribution in lipid rafts and thereby apoptosis of human colon cancer cells, so its chemosensitization of cancer cells to Doxorubicin could also be acid sphin-gomyelinase/ceramide-dependent [30,31]. Acid sphingo-myelinase transfection increased intracellular levels of ceramides in glioma cells, thereby sensitizing the cells to chemotherapy via production of reactive oxygen species [32]. This ceramide based synergistic mechanism was also shown by exogenous C6 ceramide which was able to sensitize multiple cancer lines to Doxorubicin induced apoptosis [21]. Given that Doxorubicin induced cancer cell deaths through production of endogenous ceramides as one of its mechanisms [13], it is quite conceivable that the additive effect or synergism of PPD might come from common pathway or mechanism including ceramides between two chemotherapeutic agents, although the detailed mechanisms will have to wait for further investigations in the future. In mouse xenograft model, doses of PPD tested showed anti-tumor activities comparable to Cisplatin which has shown its *in vivo* synergistic effects with Doxorubicin [33], although *in vivo* chemosensitization of PPD to Doxorubicin remains to be determined.

## Conclusions

In conclusion, we have identified the hydrolysis of membrane sphingomyelins into ceramides by neutral sphin-gomyelinase 2 as one of major mechanisms by which PPD exerts its cytotoxic effects against a spectrum of cancer cells, although further investigations are needed to fully understand them. Thus neutral sphingomyelinase 2 and its relevant mechanisms could potentially be employed in cancer chemotherapies. This investigation also demonstrated the potential of PPD as a potent adjunct to conventional anti-cancer drugs in combinational chemotherapies as well as a novel agent by itself. Thus, the clinical use of PPD as adjunct is likely to reduce toxic side effect of anti-cancer drugs and occurrence of drug resistances due to use of single agents at high doses during chemotherapies.

## Competing interests

BTGin (Daejeon, Korea) is the manufacturer who has provided the protopanaxadiol for free for conducting this scientific study and one of whose personnel, Youl Her, has been included as an author in this manuscript. Thus, the authors hereby declare that the manufacturer had no role or interest in the funding, design, conduct and interpretation of our study and findings.

## Authors’ contributions

HMK, SHO and BP conceived of the study, analyzed data and drafted the manuscript. YML was involved in mass spectrometry analysis, JSK performed confocal microscopy studies and YH provided protopanaxadiol and tested its purity and quality. JHK, BP and SHO carried out siRNA transfection and inhibition experiments. BP was involved in flow cytometry and Western Blot analyses. HMK performed the cell viability assays and *in vivo* mouse xenograft experiments. BP and HMK wrote the manuscript. All authors have read and approved the final manuscript.

## Pre-publication history

The pre-publication history for this paper can be accessed here:

http://www.biomedcentral.com/1472-6882/13/194/prepub

## Supplementary Material

Additional file 1 **Real-time PCR analysis.** Neutral sphingomyelinase 2 expression was decreased in nSMase 2 siRNA knocked down K562 (A) and HT29 (B) cells. The nSMase 2 mRNA levels were normalized against a house keeping gene, *GAPDH*. Fold changes were calculated relative to control. *, p < 0.05; **, p < 0.01; ***, p < 0.001 for control (vehicle) are considered as significant. Click here for file

Additional file 2 **Protopanaxadiol (PPD) exhibits potent cytotoxic effects on other cancer cells than K562 in XTT assay.** ACHN (A), PC-3 (B) NCI-H23 (C) and Caki-1 (D) cells were treated with PPD at 0, 25 or 50 μM for 24 and 48 hr, then their viabilities were assessed using XTT assay. *, p < 0.05; **, p < 0.01; ***, p < 0.001 for control (vehicle) are considered as significant. Click here for file

Additional file 3 **Protopanaxadiol (PPD) exhibits potent cytotoxic effects on other cancer cells than K562 in SRB assay.** ACHN (A), PC-3 (B) NCI-H23 (C) and Caki-1 (D) cells were treated with PPD at 0, 25 or 50 μM for 24 and 48 hr, then their viabilities were assessed using SRB assay. *, p < 0.05; **, p < 0.01; ***, p < 0.001 for control (vehicle) are considered as significant. Click here for file
