# Supplementary figures and images for: Neutral sphingomyelinase 2 modulates cytotoxic effects of protopanaxadiol on different human cancer cells
Source: BMC Complement Altern Med. 2013 Jul 27;13:194. doi: 10.1186/1472-6882-13-194 (PMC3729373; doi:10.1186/1472-6882-13-194)

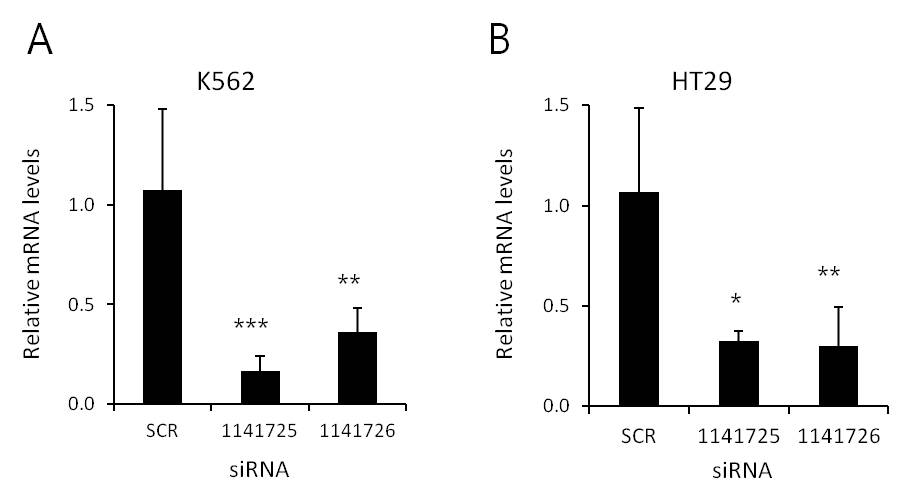

Supplement: Additional file 1 — Real-time PCR analysis. Neutral sphingomyelinase 2 expression was decreased in nSMase 2 siRNA knocked down K562 (A) and HT29 (B) cells. The nSMase 2 mRNA levels were normalized against a house keeping gene, GAPDH. Fold changes were calculated relative to control. *, p < 0.05; **, p < 0.01; ***, p < 0.001 for control (vehicle) are considered as significant. [file 1472-6882-13-194-S1.jpeg]

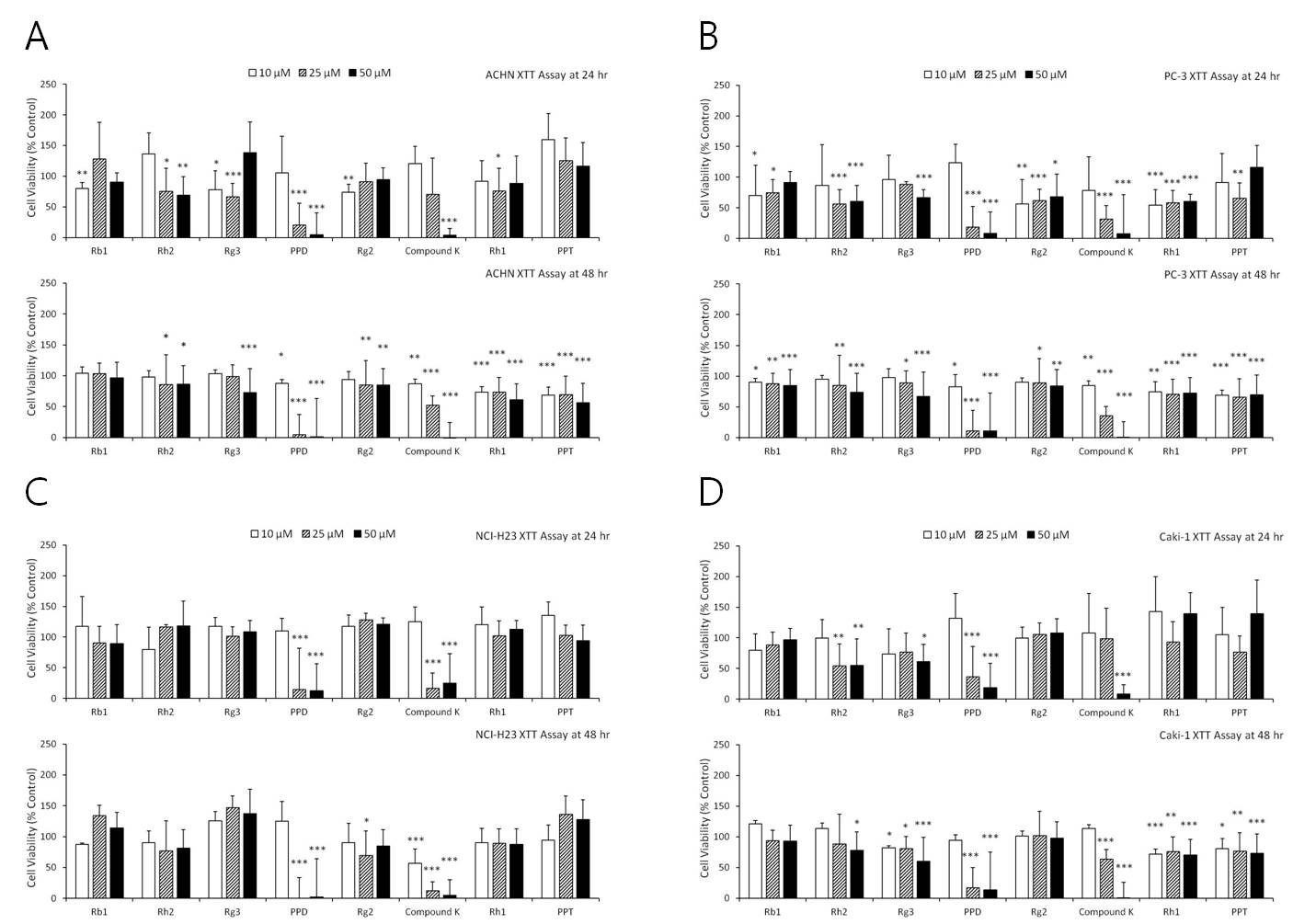

Supplement: Additional file 2 — Protopanaxadiol (PPD) exhibits potent cytotoxic effects on other cancer cells than K562 in XTT assay. ACHN (A), PC-3 (B) NCI-H23 (C) and Caki-1 (D) cells were treated with PPD at 0, 25 or 50 μM for 24 and 48 hr, then their viabilities were assessed using XTT assay. *, p < 0.05; **, p < 0.01; ***, p < 0.001 for control (vehicle) are considered as significant. [file 1472-6882-13-194-S2.jpeg]

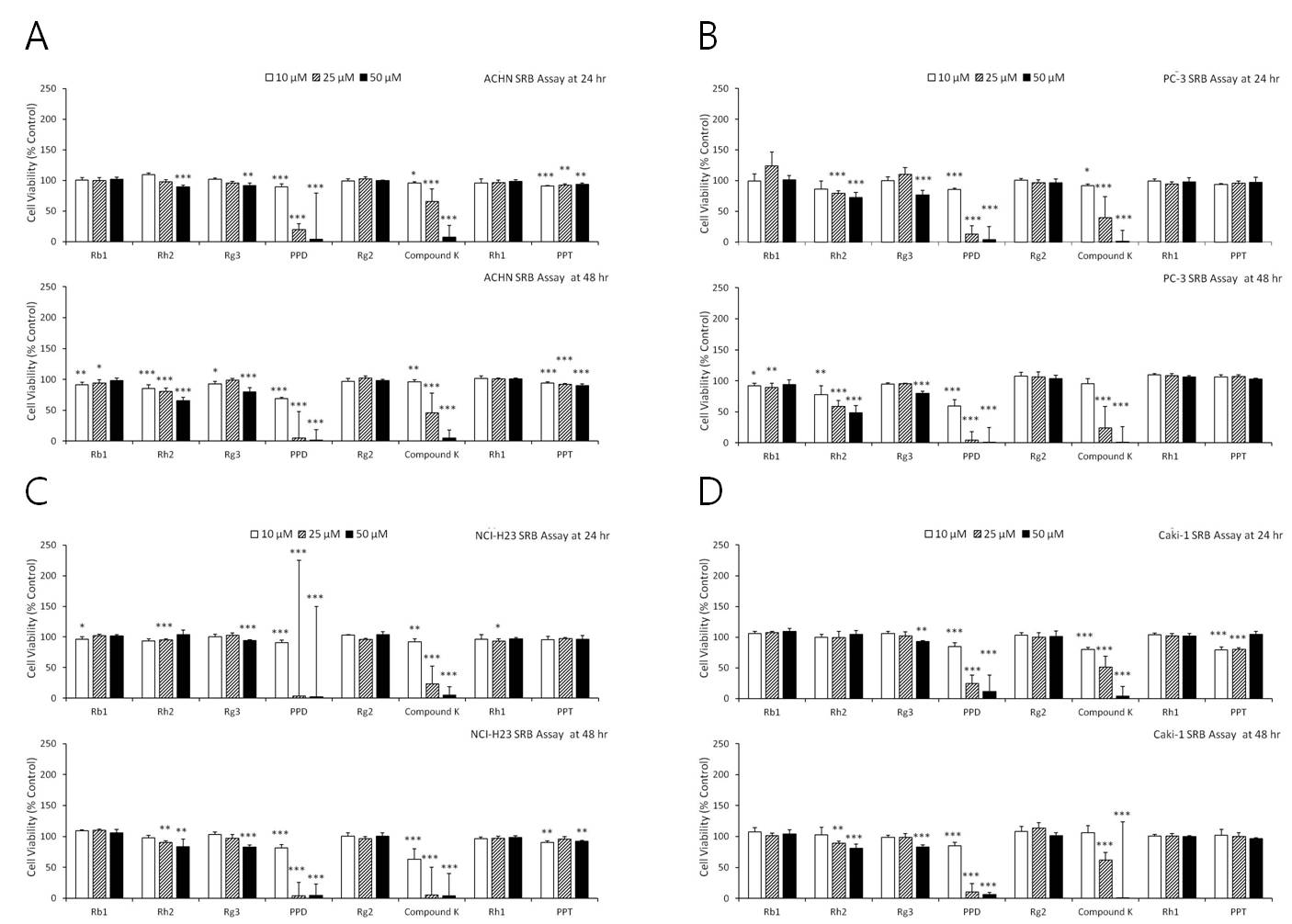

Supplement: Additional file 3 — Protopanaxadiol (PPD) exhibits potent cytotoxic effects on other cancer cells than K562 in SRB assay. ACHN (A), PC-3 (B) NCI-H23 (C) and Caki-1 (D) cells were treated with PPD at 0, 25 or 50 μM for 24 and 48 hr, then their viabilities were assessed using SRB assay. *, p < 0.05; **, p < 0.01; ***, p < 0.001 for control (vehicle) are considered as significant. [file 1472-6882-13-194-S3.jpeg]
